# Analysis of Large Seeds from Three Different *Medicago truncatula* Ecotypes Reveals a Potential Role of Hormonal Balance in Final Size Determination of Legume Grains

**DOI:** 10.3390/ijms17091472

**Published:** 2016-09-08

**Authors:** Kaustav Bandyopadhyay, Orhan Uluçay, Muhammet Şakiroğlu, Michael K. Udvardi, Jerome Verdier

**Affiliations:** 1 The Samuel Roberts Noble Foundation, Plant Biology Division, 2510 Sam Noble Parkway, Ardmore, OK 73401, USA; kaustav_01@yahoo.co.in (K.B.); orhanulucay@gmail.com (O.U.); mudvardi@noble.org (M.K.U.); 2 Kafkas University–Faculty of Engineering and Architecture, Central Campus, Kars 36100, Turkey; msakiroglu@gmail.com; 3 Shanghai Center for Plant Stress Biology, Shanghai Institutes of Biological Sciences, Chinese Academy of Sciences, 3888 Chenhua Road, Shanghai 201602, China

**Keywords:** legume grains, abscisic acid, auxin, cell division, embryogenesis, *Medicago truncatula*, seed size

## Abstract

Legume seeds are important as protein and oil source for human diet. Understanding how their final seed size is determined is crucial to improve crop yield. In this study, we analyzed seed development of three accessions of the model legume, *Medicago truncatula*, displaying contrasted seed size. By comparing two large seed accessions to the reference accession A17, we described mechanisms associated with large seed size determination and potential factors modulating the final seed size. We observed that early events during embryogenesis had a major impact on final seed size and a delayed heart stage embryo development resulted to large seeds. We also observed that the difference in seed growth rate was mainly due to a difference in embryo cell number, implicating a role of cell division rate. Large seed accessions could be explained by an extended period of cell division due to a longer embryogenesis phase. According to our observations and recent reports, we observed that auxin (IAA) and abscisic acid (ABA) ratio could be a key determinant of cell division regulation at the end of embryogenesis. Overall, our study highlights that timing of events occurring during early seed development play decisive role for final seed size determination.

## 1. Introduction

Increasing seed yield has been one of the major objectives for plant biologists in the last decades. Seed size and seed number are two complementary important agronomic traits in yield determination for crop species, considering that seed is the first source of calories in human and animal diets. Since the beginning of crop domestication, crop species have been selected based on the size of their seeds. Larger seeds accumulate more storage molecules, which benefit food production. From a plant perspective, seed size is also crucial in evolutionary fitness. Indeed, larger seeds accumulate more reserve, which will allow seeds to germinate at higher rate and be more tolerant to abiotic stresses. Conversely, smaller seeds are more efficient in dispersing and colonizing new territories [1].

In the past years, several mechanisms have been described as components of the seed size determination [2,3]. Final seed size is the result of coordinated growth of three tissues: the embryo, the endosperm and the seed coat [4]. These tissues derived from maternal tissues (i.e., seed coat) or from different fertilization events (i.e., one sperm cell fertilizes the diploid central cell to form the triploid endosperm and the second sperm cell fertilizes the egg cell to form the diploid embryo). The final seed size is mainly contributed by the size of the embryo in dicotyledonous plants. In order to generate larger seeds, the embryo undergoes a series of cell division and cell expansion. It has been demonstrated that these events occur at specific timeframes during the seed development: cell division is mainly associated with the embryogenesis phase, whereas the cell expansion is more associated with the seed-filling phase resulting in a mature seed [5]. The embryogenesis is a morphogenetic process, tightly regulated by a genetic program that will define the final number of cell. During this process, the zygote will undergo series of cell division, which will lead to different developmental stages of the embryo, (i.e., globular, heart, torpedo, bent cotyledon and finally mature embryo) [5]. According to previous reports, seed size is controlled by various genes involved in different mechanisms [6]). A non-exhaustive list consists of transcription factors such as *APETALA2* (*AP2*) [7,8] or *MINISEED3* (*MINI3*); [9]), cytochrome P450 gene such as *KLU* [10], G proteins [11] and E3 ligases such as *BIG* *BROTHER* (*BB*; [12]) or *DA2* [13].

Legumes are the second most economically important plant family after cereals. Legume seeds represent a major source of plant proteins. With the increasing demand for proteins, understanding how legume seeds regulate their size has been a crucial question to address. A large variation of seed size across and within species exists in legumes, which provides important genetic resources to understand how species regulate their seed size. For instance, from the smallest to the largest major legume seeds, we could list alfalfa (*Medicago sativa*), then lentils (*Lens culinaris*), mung bean (*Vigna radiata*), garden pea (*Pisum sativum*), soybean (*Glycine max*) and faba bean (*Vicia faba*). *Medicago truncatula* has emerged as a model for studying legume biology in recent years. This species has been widely used in functional genomic studies mainly due to its relatively small genome compared to other legumes, which was sequenced in 2011 [14], its capacity of in vitro regeneration for transformation [15], the availability of mutant populations [16,17,18] and the development of other tools such as the gene expression atlas [19].

During domestication, seed size was a major determinant of seed yield and a large variation of this trait still exists within species. Thus, germplasm collections can be exploited to investigate this trait. Seed yield results from two additive components: the size of individual seed and the number of seeds per plant. In this study, we focused on the events occurring during the seed development leading to seed size determination, without investigating remobilization processes from plant to seed that potentially lead to change in seed number. We used the genetic variability of three different *Medicago* accessions to decipher the differences between normal and larger seeds. We analyzed both the mature and developing seeds in terms of growth pattern, embryo development, cell expansion, cell proliferation and finally hormonal homeostasis.

## 2. Results

### 2.1. Identification of Medicago Accessions with Larger Seeds and Different Growth Patterns

**Figure 1:**
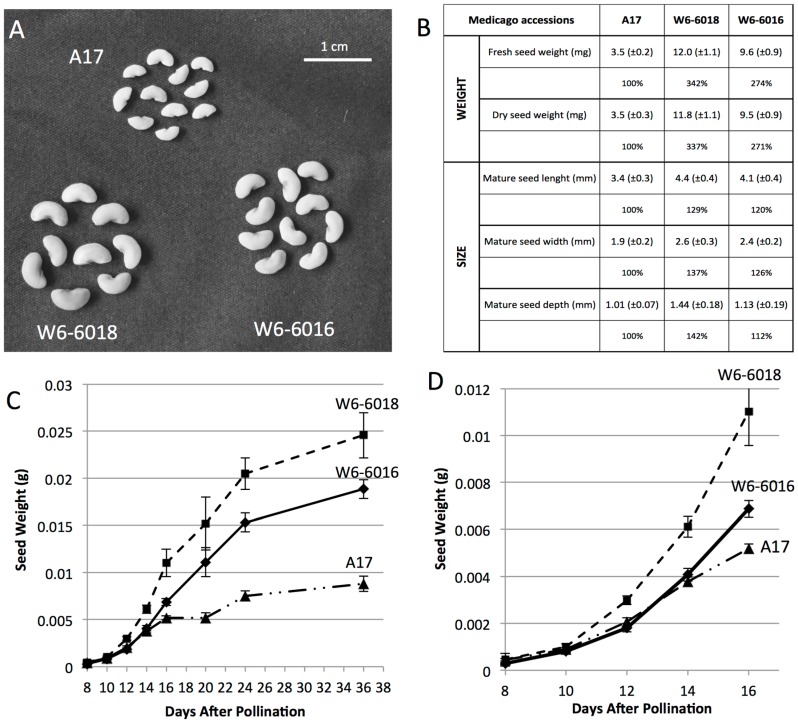
Seed growth patterns of different accessions of *Medicago*. (**A**) Pictures of mature seeds of the three accessions; (**B**) Mature seed weight and size of the different accessions from fresh and dry seeds. Values are averaged for one mature seed. Standard deviations are indicated between brackets. The percentage of weight/size increase with respect to the reference A17 is also indicated for each accession (*n* > 50); (**C**) Average fresh weight of one seed of three *Medicago* accessions (A17, triangles; W6-6016, diamonds and W6-6018, squares) are plotted against their developmental stages (days after pollination) (*n* = 100); (**D**) Magnification of figure 1C between 8 to 16 DAP.

From the Germplasm Resource Information Network (GRIN USDA, ARS, National Genetic Resources Program, http://www.ars-grin.gov), two *Medicago* *truncatula* accessions were selected for their large seed size (i.e., around one gram for 100 seeds). W6-6018 (PI660406) is an accession collected from Germany and annotated as wild material. W6-6016 (PI660404) is an accession collected from Cyprus and annotated as cultivated material. To compare with these *Medicago* accessions, we used the line Jemalong A17 (PI 670016) [14], which is considered as the reference line for most of genomics and genetics studies [20] and display an average seed weight of 300 mg for 100 seeds. After growing these different accessions under the same conditions, we confirmed that W6-6018 and W6-6016 plants produced larger mature seeds than the reference A17. We observed that mature seeds from W6-6018 and W6-6016 were, respectively, 3.4- and 2.7-fold heavier than A17 (Figure 1A,B). Fresh mature seeds were dried at 80 °C for 48 h and the percentage increase of dry seed weights of different accessions were similar to those of fresh mature seeds indicating that the water uptake or desiccation processes were not responsible of the differences in weight (Figure 1B). We also measured the size of mature seeds in order to determine which of the three-dimensional axes (i.e., length, width and depth) of the seeds were the most affected by the increase of size. We found that all the three dimensions displayed increased size, which resulted in the increase of final seed weight (Figure 1B). Then, we measured the fresh weights of developing seeds from all three different accessions: W6-6016, W6-6018 (both big seeds) and A17 (as the reference), starting from eight days after pollination (DAP) to 36 DAP. We noticed different growth patterns for the three accessions. The seeds of W6-6018 displayed similar weight compared to A17 until 10 days, but then significantly increased and remained bigger throughout the development (Figure 1C). W6-6016, on the other hand, were smaller than A17 until 12 DAP and then overtook the growth of A17 by 14 DAP (Figure 1D). These growth patterns indicated that the regulatory mechanism(s) conferring larger seeds occurred between 10 and 12 DAP for W6-6018, and 14 and 16 DAP for W6-6016, thus during the embryogenesis phase.

### 2.2. Embryo Development Was Delayed in the Large Seed Accessions

**Figure 2:**
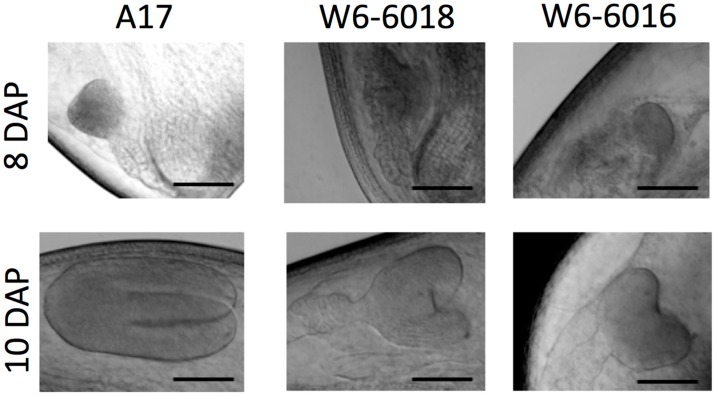
Embryo development of the different accessions. Differential interference contrast microscope (DIC) images of growing embryos from different accessions of *Medicago* (A17, **left panel**; W6-6016, **middle panel**; and W6-6018, **right panel**) taken at 8 DAP (**upper panel**) and 10 DAP (**lower panel**). Scale bars = 0.5 mm.

To get a detailed description of the embryogenesis during the seed development between these accessions, we treated seeds with chloral hydrate to clear them and then used a differential interference contrast microscope (DIC) to visualize the different stages of embryo development at different time points. To compare between accessions, we first analyzed the embryo growth of the A17 reference line. At 8 DAP, most of the A17 embryos were at the early heart stage, whereas at 10 DAP, embryos reached the torpedo or late torpedo stage, as described in previous studies [21,22]. In comparison to A17, embryo development in the two large seed accessions appeared to be slower by at least a day or two. At 8 DAP, the embryos of W6-6016 and W6-6018 were still at the globular stage, morphologically preceding the heart stage of A17 embryos (Figure 2). By 10 DAP, embryos of the two large seed accessions only reached the heart stage for W6-6016 and the early heart stage for W6-6018 (Figure 2), whereas A17 embryos were more advanced at the torpedo stage. By 12 DAP, all the accessions displayed fully developed embryos with development of cotyledons (i.e., cotyledon stage). However, cotyledons of the large seed accessions appeared bigger than of A17. These results suggest that based on visual observations from the DIC images, A17 reached cotyledon stage faster than other accessions, which impacts the size of the whole embryo (Figure 2).

### 2.3. The Increase of Mature Seed Size Was Due to Increase in Cell Division but Not Cell Expansion

**Figure 3:**
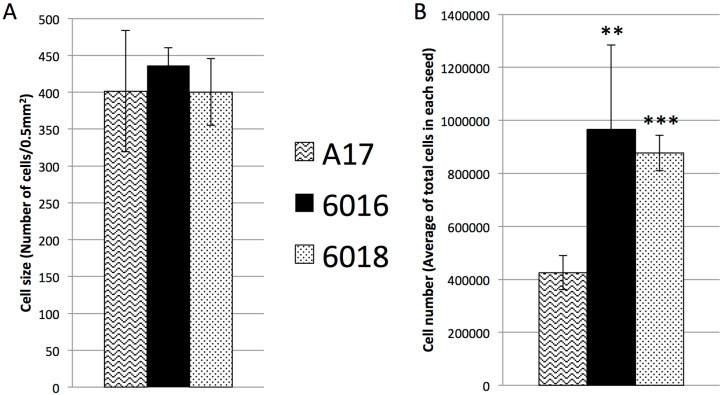
Cell size and cell number in mature seeds of the three accessions. (**A**) Cell size measured as number of cells counted in frozen sections of mature seeds in a specific area (0.5 mm^2^) (*n* = 6); (**B**) Average of total number of cells obtained from single mature seeds (excluding seed coat) of the three different accessions using a haemocytometer cell (*n* = 16). Statistical differences resulting from *t*-test are indicated with **: *p*-value < 0.01 and ***: *p*-value < 0.001.

The reasons behind the accessions displaying larger seeds than A17 could be an increase of the cell number and/or an increase of the cell expansion in embryo. To find the proper answer, we prepared frozen microscopic sections of mature seeds from all three accessions and stained them with Toluidine blue. By comparing microscopic sections at the same magnification, we counted the number of cells in a given area of embryos to calculate the cell size, reflecting the cell expansion. By counting the number of cells in 0.05 mm^2^, we did not observe any significant difference across the accessions (Figure 3A). Therefore, the final cell size in the mature embryos of all the accessions was similar and does not explain the difference in seed size. Since all three accessions displayed similar cell size in mature seeds, we checked the cell number by counting the total number of cells per single mature seed from all three accessions. The seeds were homogenized and digested uniformly into a suspension of cells using an enzymatic solution after the removal of the seed coat. The total number of cells in each seed was determined by using a haemocytometer cell counter. At maturity, *Medicago* seeds contain a residual endosperm representing less than 10% of the final seed size. After seed coat removal, most of digested cells were embryo cells [23]. Our result showed a statistically significant increase of the total cell numbers in both of the large seed accessions, with respect to mature A17 seeds (Figure 3B). This result suggests that the mature seed size in the large seed accessions is determined by a higher number of cells in embryos.

**Figure 4:**
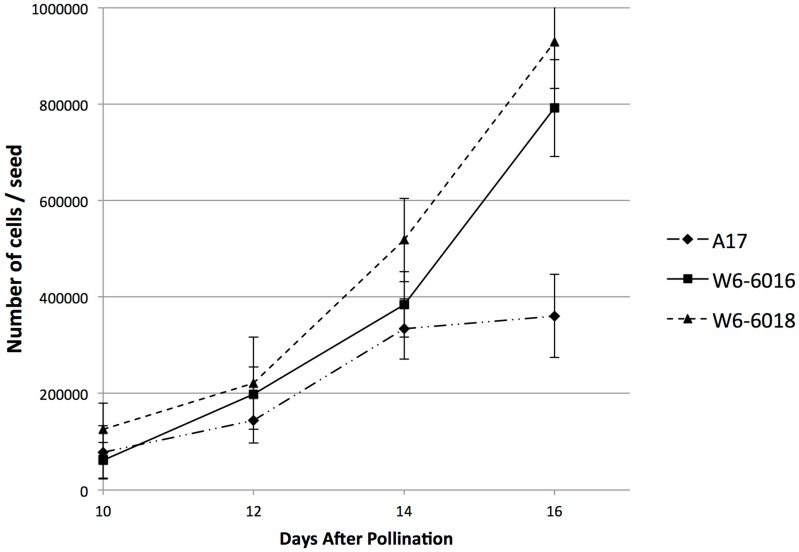
Cell proliferation during 10–16 DAP of seed development. Average total number of cells calculated to be present in a single seed of each of the accessions: A17 (**diamonds**), W6-6016 (**squares**) and W6-6018 (**triangles**). Standard deviations are indicated.

According to this result and our previous observations that the determination of the final seed size at maturity occurs at earlier stages of seed development, we analyzed the embryo cell number during the embryogenesis. We performed the same analysis of cell counting during embryogenesis, from 10 to 16 DAP, to resolve the timing of size determination. As shown in Figure 4, cell proliferation in A17 ceased by 14 DAP, whereas it continued until 16 DAP in the large seed accessions. The pattern of cell proliferation was similar to the pattern of weight gain by the seeds at these time points (compare Figure 1D and Figure 4). At 10 DAP, we observed a similar number of cells between all accessions, despite the delay in embryogenesis (i.e., A17 embryos were at the torpedo stage and W6-6016 and W6-6018 embryos were at the heart stage). From 12 DAP, we noticed a difference in cell proliferation between the large accession and A17 seeds. W6-6016 and W6-6018 displayed a higher cell proliferation rate than A17 at 14 and 16 DAP, respectively (Figure 4). Interestingly, we observed a stop in cell division at 14 DAP for A17 seeds, whereas W6-6018 and W6-6016 cells continued to proliferate afterwards.

### 2.4. Change in ABA to IAA Ratio Correlates with the Variation of Seed Size

**Figure 5:**
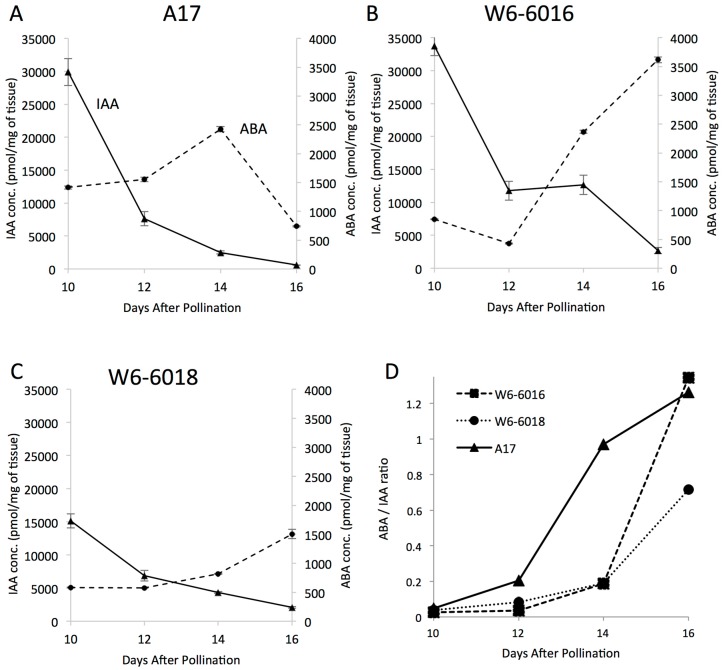
IAA and ABA profiles of the three accessions. (**A**–**C**) IAA (solid line, *Y*-axis on the **left**) and ABA (dashed line, *Y*-axis on the **right**) levels of A17 (**A**); W6-6016 (**B**); and W6-6018 (**C**) are plotted against the developmental age (DAP) of the seed; (**D**) The corresponding ratios of ABA to IAA from the above figures are plotted against the developmental age of the seeds (DAP) of A17 (**triangles**), W6-6016 (**squares**) and W6-6018 (**circles**). Standard deviations are indicated in the different panels.

The growth kinetics and embryo development studies presented in this study suggested that the difference in seed size among the accessions was due to events occurring between 10 and 14 DAP. According to our data, the increase in seed size was mainly due to a delay in embryogenesis and thus an extended period of cell division at the end of embryogenesis. Therefore, we quantified total auxin (IAA) and abscisic acid (ABA) during embryogenesis in the different accessions. IAA and ABA were chosen because they are respectively the key hormones to control the pattern formation in embryogenesis and the promotion of reserve accumulation during the subsequent seed filling phase [24,25]. Using liquid chromatography coupled to mass spectrometry, we quantified these phytohormones between 10 and 16 DAP to correlate their content with the difference in seed size or cell division (Figure 5). First, we analyzed the concentrations of IAA or ABA in developing seeds and we did not observe significant difference in developing seeds of A17 and W6-6016 until 16 DAP (Figure 5A,B). Seeds from W6-6018 accumulated smaller amounts of IAA and ABA (Figure 5C) but without any correlation with cell proliferation. Thus, individual concentrations of these phytohormones were not linked to the difference in seed size or cell division. Therefore, we compared the accumulation patterns of these phytohormones in the three accessions (Figure 5A–C). We observed that in the three accessions, IAA content was decreased from 10 to 16 DAP with almost no IAA left at 16 DAP. On the other hand ABA content was increasing within the same period of time. Moreover, a major difference between A17 and the large seed accessions was the ABA content after 14 DAP, which decreased in A17, but was still increasing in W6-6016 and W6-6018. The difference in ABA content at the later stage has led us to compare the ratio of ABA to IAA accumulation between 10 and 16 DAP (Figure 5D). We observed a big difference in the patterns, especially how the ratio of ABA to IAA changes between large seed accessions and A17. The distinct profiles of ABA/IAA ratio between A17 and the other accessions displayed negative connections with the kinetics of seed growth (Figure 1D) and the cell proliferation rate (Figure 4). Indeed, at 10–12 DAP, the three accessions displayed similar ABA/IAA ratio, similar weight and similar cell proliferation. Then, at 14 DAP, when we observed a change in seed growth and cell proliferation, we similarly observed a change in ABA/IAA ratio between A17 and the two large seed accessions, which coincided with the difference in cell proliferation rates between the accessions (Figure 4). Finally, at 16 DAP, we detected a difference in ABA/IAA ratio between W6-6016 and W6-6018, which coincided with the difference in cell proliferation between these two large seed accessions (i.e., as W6-6018 produced even larger mature seeds than W6-6016). Therefore, our hypothesis is that cell division in cotyledons can continue until the level of IAA is sufficiently lowered and the level of ABA is increased to a point where it can inhibit the cell cycle. For instance, if we consider the ABA/IAA ratio as a regulator of the cell cycle, we noticed that A17 reached a ratio of 1 at 14 DAP, W6-6016 around 16DAP and W6-6018 after 16 DAP, which correlate with the cell proliferation that decreased at 14 DAP for A17 but continued to increase for W6-6016 and W6-6018 and also correlate with the final seed size of the three accessions. Even if the ABA/IAA is, indeed, a regulator of the final seed size, their effect on cell proliferation or cell cycle has to be mediated through other hormones. We tested two common forms of cytokinins in plants, tZR and iPR. We did not find a correlation between the cell proliferation and tZR concentration throughout the seed developmental stages. However, we did observe a correlation between the concentration of iPR and the cell proliferation, which could be an active form of cytokinins during Medicago seed development and explain the increase of cell proliferation in W6-6016 and W6-6018 (Figure S1).

## 3. Discussion

### 3.1. Increase of Seed Size as a Result of Increase of Cell Division during Embryogenesis

In this study, we analyzed two accessions of *Medicago truncatula*, which were selected because of their significantly larger seeds than the reference accession, A17 (2.7-fold for W6-6016 and 3.4-fold for W6-6018). Seed size in monocots is attributed to the extent of endosperm growth [26,27]. In dicot seeds such as those of legumes (garden pea), the volume is mostly made up of cotyledons and it has been shown that cell number and size of cotyledon cells are directly associated to final seed size [28]. Any difference in cotyledon (hence seed) size arises from one of the following two reasons: the larger seeds contain more cells, implicating a difference in cell division, or larger cells, implicating cell expansion (or a combination of both phenomena). It has been reported that active cell division occurs early during seed development and stops before seed maturation, while cell expansion mainly occurs during seed maturation [5]. In the large seed accessions, we observed that the size difference between mature seeds can be explained by a change in cotyledon cell number (Figure 3), thus the rate of cell division. Previous report on *Medicago* *truncatula* (reference accession A17) embryogenesis showed that the cell division in the embryo peaks at the heart and torpedo stages and diminishes after the cotyledon stage is reached [29]. Our Differential interference contrast microscope (DIC) imaging data with A17 embryos are consistent with these results showing that embryo development of the two large seed accessions was delayed at the heart and torpedo stages, potentially allowing more cell division in comparison to A17. By analyzing cell proliferation during embryogenesis, we confirmed that the large seed accessions displayed a longer and higher rate of cell division. In A17, cell proliferation was decreased around 14 days after pollination (DAP). But W6-6016 and W6-6018 continued to generate more cells after 16 DAP (Figure 4). Interestingly, another example of slower embryo development leading to larger seed size was described in *Arabidopsis* *apetalla2* mutant (*ap2*), which displayed an initially delayed embryogenesis and finally larger seeds with respect to WT [30]. For *ap2* mutant, this delay allows a longer embryo growth with extended cell division activities and ultimately larger seeds. This mechanism is probably similar in W6-6016 and W6-6018, which were characterized by a delay in early embryogenesis and a longer cell proliferation rate than A17 (Figure 4).

Even if seed size determination is a result of cell division and cell expansion, we did not observe a change in cell size area of mature seeds, suggesting that cell division was the major factor determining seed size in the large seed accessions chosen for this study. This predominance of cell division over cell expansion in seed size determination has also been observed in *sbt1* and *dash* *Medicago* mutant lines [22,31], suggesting that early cell division is indeed a critical factor determining the final seed size.

### 3.2. Phytohormones as Regulators of Cell Division

The role of auxin in cell division and plant development of various organs is well established [32,33] and the seed is no exception. However, during seed development, the role of auxin has been mostly described on the acquisition of embryo polarity and patterning [34]. Very few attempts to correlate auxin with cell division in developing embryo have been made so far. *Arabidopsis* *ARF2*, an auxin responsive gene, has been described as a negative regulator of cell division. Mutation of *ARF2* causes an increase of seed size, which suggests auxin impacts the final seed size by inhibition of cell division in specific seed cell-type via *ARF2* [35]. In *Medicago*, It has been shown that mutation of a transcription factor gene, named *DASH*, affects proper embryo development and auxin homeostasis in pods leading to smaller mature seeds [22]. These published results prompted us to compare absolute concentrations of IAA and IAA accumulation profiles between 10 and 16 DAP across the accessions. But we did not observe any correlation between IAA concentrations with cell division or final seed size. Indeed, auxin concentration alone may not be enough to cease cell division. Auxin acts as an initiation or ‘turn on’ switch and acts through secondary regulators. Just decreasing auxin concentration (i.e., removal of the turn on switch) may not lower the other hormones. Considering this hypothesis, a second ‘turn off’ switch is potentially necessary to accurately define the time frame of cell division. The role of ABA at the end of embryogenesis is well established as a promoter of reserve accumulation, thus acting as a switch from the embryogenesis to enter into the seed filling phase [36]. But other literatures also indicate a role of ABA even before seed filling [37,38]. According to these reports, a small amount of ABA produced from the maternal source is accumulated before cell division arrests. This fact probably strengthens the idea of ABA as the second turn off switch for cell division. While some plants have two peaks of ABA during seed development, others have only one broad peak [39]. In legumes, this varies a lot from species to species [40] and nothing is known about *Medicago*. It has already been proposed that the role of ABA is to ensure the end of cell division before the maturation [39]. Mechanistically, it has been shown that ABA can inhibit cell division by two ways. First, ABA induces the expression of a cyclin-dependent kinase inhibitor (*ICK1*) that would lead to cell cycle arrest at the G1/S transition [41]. ABA can also control the final seed size in a second mechanism, by activating ubiquitin receptor DA1, which is an inhibitor of cell proliferation [2].

According to our results and related studies described previously, we hypothesize that a high and extended ABA to IAA ratio during the end of embryogenesis (10–16 DAP) could define cut-off of active cell division and correlate with the final seed size. This hypothesis is supported by the similarity between the ABA/IAA ratio and cell proliferation curves occurring between 10 and 16 DAP (Figure 4 and Figure 5). When ABA/IAA ratio was low (i.e., more auxin than ABA), we observed an active cell proliferation. When a critical ABA/IAA ratio is reached, we observed an arrest of cell division in A17, whereas W6-6016 reached this ratio around 16 DAP and W6-6018 at later stage than 16 DAP, which led to the smallest to largest seed phenotype (i.e., respectively A17, W6-6016 and W6-6018). Our hypothesis is also supported by two recent studies from *Medicago*. First, [42] used in vitro auxin treatment of immature *Medicago* seed and reported that external auxin treatment on 8, 10, and 12 DAP *Medicago* seeds increased the weight and size of seeds by promoting cell division. A second report by [22] showed that a change in IAA homeostasis during the end of embryogenesis impaired the embryo cell division rate, the timing of embryogenesis and ultimately the final seed size.

If our hypothesis is true and ABA/IAA ratio potentially regulates final seed size, their effect would be mediated through other hormones, such as cytokinins, gibberellic acid (GA) and brassinosteroids (BR). In this study, we correlated the cell proliferation rate of the different accessions with the concentration of iPR, which could be one of the active forms of cytokinins in *Medicago* seed development (Figure S1). Indeed, the level of cytokinins increases during embryo development in many seeds including legumes [43]. Nonetheless, a recent report has shown the presence of very little or no biologically active cytokinin in some legumes such as chickpea, which is phylogenetically close to *Medicago* [40]. Regarding GA and BR, their role in cell division and organ development has also been established but they were not tested. Gibberellins are well known as potential regulator of cell division. But in case of seeds, reports of the action of GAs is mainly restricted to seed germination [44]; for review, see [45]. Finally, recent studies have established a role of brassinosteroids in determining seed size, even if so far its role has been more related to cell expansion [46]. Nonetheless, not much work has been done in *Medicago* and roles and occurrence of these hormones vary from legume to legume, as we have seen earlier [40]. Our effort can be considered as an initiation of many such studies to come.

## 4. Experimental Section

### 4.1. Plant Material and Growth Conditions

Large seed *Medicago* accessions used in this study (W6-6018 and W6-6016) were requested from the Germplasm Resource Information Network (GRIN USDA, ARS, National Genetic Resources Program, http://www.ars-grin.gov). Seeds of different *Medicago* accessions were scarified with concentrated sulfuric acid, rinsed, sterilized with 20% sodium hypochlorite, and vernalized at 4 °C for 3 days on moist sterile filter paper. Germinated seedlings were transplanted to pots containing soil and placed in a greenhouse with the following conditions: 16 h/8 h light/dark regime, 200 μE·m^−2^·s^−1^ light irradiance, 24 °C temperature and 40% relative humidity. Fully opened flowers were tagged as described in [23], the day of tagging was considered as 1 day after pollination (1 DAP) since self-pollination in *Medicago truncatula* occurs inside the closed flower. The pods were collected at different time points and seeds were dissected for further analyses.

### 4.2. Fresh and Dry Weight Measurement

The dissected seeds from different time points were weighed, as set of ten seeds, to calculate the average weight per seed. Ten readings (i.e., 100 seeds in total) were taken at each developmental stage for statistical analysis. The same sets of seeds were, then, dried at 80 °C for 48 h. The dry weight measurements were taken as described above. Mature seeds size (i.e., length, width and depth) was measured using a dial caliper and averaged from individual measurement of more than 50 seeds.

### 4.3. Clearing of Seeds for DIC Imaging

Freshly dissected seeds were clarified by a solution of Chloral hydrate in 30% Glycerol (2.5 g chloral hydrate in 1 mL of 30% glycerol). The seeds were fixed in a solution of 9 parts ethanol: 1 part acetic acid for 2 h. They were then washed twice with 90% ethanol for 30 min each wash. Seeds were then placed into the chloral hydrate solution and kept in dark overnight. The seeds were mounted on a glass slide in 30% glycerol. For each ecotype, four seeds were dissected from four different pods, collected from four different plants.

### 4.4. Counting Total Number of Cells in Mature Seeds

The seeds were scarified and soaked in water as mentioned above. The seed coats were then removed to mainly isolate the embryo. Four seeds were chopped with a blade and 1 mL of “cell wall digesting enzyme mix” was added (0.45 M sorbitol, 10 mM MgCl_2_, 1 mM KH_2_PO_4_, 20 mM MES pH 5.6, 0.4% Macerozyme R10, cellulase Onozuka R10 1% (phytotechnology)). The samples were incubated at 37 °C for 24 h, with regular gentle vortexing. 0.5 mL of the ‘cell wall digesting enzyme mix’ was added after 24 h and the incubation was carried out for another 24 h, until we obtained a homogeneous mixture of dispersed cells. The cells were then diluted to 2 mL and 50 μL was used to count the cells in a haemocytometer counter chambered slide (Fisher biotech, Pittsburg, PA, USA). The total number of cells in 2 mL was calculated from this value, which corresponded to the total number of cells from four seeds. This was divided by 4 to get the number of cells in each seed. For each ecotype, sixteen seeds were collected from four different pods, collected from four different plants.

### 4.5. Counting Number of Cells in a Given Area (Cell Size)

The mature seeds were scarified with H_2_SO_4_ for 10 min followed by repeated washes with water. The seeds were soaked in water for 16 h and then in PBS. After 2 h, the seeds were placed in a solution containing 20% sucrose diluted in PBS. They were further incubated for 16 h before being embedded to OCT embedding solution (Electron Microscopy Science, Hatfield, PA, USA). 15 μm sections were made in a cryo-microtome (Leica, Buffalo Grove, IL, USA). The slides were stained in toluidine blue solution followed by washes with water. The images were taken at same magnification, from similar locations and plains of the seeds. The cell number was calculated by counting the number of cells in embryo from a specific area (0.05 mm^2^). For each ecotype, six seeds (representing different pods and different plants) were sectioned.

### 4.6. Phytohormone Measurement

Fresh samples were ground in liquid nitrogen and 50 mg of the powders were used per sample for hormone extraction. We started grinding with around 200 mg of fresh tissue, which is approximately 100–200 seeds (since each seed weighs around 1–3 mg at 10–14 DAP depending on the ecotype). These 100–200 seeds were collected from 20–25 different pods from the same plant. Four such plants were maintained to get four biological replicate of the whole experiment. Therefore, each reading at the LC-MS/MS is an average of at least 100 seeds and we have four such readings. The final data are expressed as an average of those four readings.

The samples (25 mg powder) were extracted in 1 mL of extraction buffer (isopropanol, water and hydrochloric acid in a 2:1:0.002 *v*/*v* mixture). 50 pmol of each of the labeled standards (d5-IAA, d6-ABA) were then added to the samples. After one hour of extraction, 0.5 mL of dichloromethane was added to the samples. The samples were kept in a shaker for 30 minutes at 4 °C and then were centrifuged at 2500× *g* for 30 min. After centrifugation, one mL sample from the bottom layer was transferred into a fresh glass vial and dried under nitrogen flow. The dried residues were re-dissolved in 0.1 mL methanol followed by the addition of one mL of 1% acetic acid. The samples were filtered through Oasis HLB extraction cartridges (Waters, Milford, MA, USA). The columns were pre-equilibrated with 1 mL acetonitrile followed by 1 mL methanol and then with 2 mL 1% acetic acid. The samples were passed through the columns and were washed with one mL of 1% acetic acid and were then eluted with 1.8 mL elution buffer (80% methanol and 1% acetic acid). The samples were dried under nitrogen flow and re-dissolved in 25 μL of methanol. A volume of 25 μL of 1% acetic acid was then added to each sample before injection into the LC-MS/MS system (A reverse-phase 1.7 μm UPLC BEH C18, 2.1 × 150 mm column (Waters) coupled with Agilent technology triple quad system (Santa Clara, CA, USA)). The hormones were identified using the standards as reference and quantified by MassHunter quantitative analysis software using Multiple Reaction Monitoring (MRM) method.

## Supplementary Materials

Supplementary materials can be found at www.mdpi.com/1422-0067/17/9/1472/s1.
