# Supplementary material for: Analysis of Large Seeds from Three Different Medicago truncatula Ecotypes Reveals a Potential Role of Hormonal Balance in Final Size Determination of Legume Grains
Source: Int J Mol Sci. 2016 Sep 8;17(9):1472. doi: 10.3390/ijms17091472 (PMC5037750; doi:10.3390/ijms17091472)
Supplement: Supplementary file 1 [file ijms-17-01472-s001.pdf]

Int. J. Mol. Sci. **2016**, *17*, 1472; doi:10.3390/ijms17091472

Supplementary Materials: Analysis of Large Seeds from Three Different *Medicago truncatula* Ecotypes Reveals a Potential Role of Hormonal Balance in Final Size Determination of Legume Grains

Kaustav Bandyopadhyay, Orhan Uluçay, Muhammet Şakiroğlu, Michael K. Udvardi and Jerome Verdier

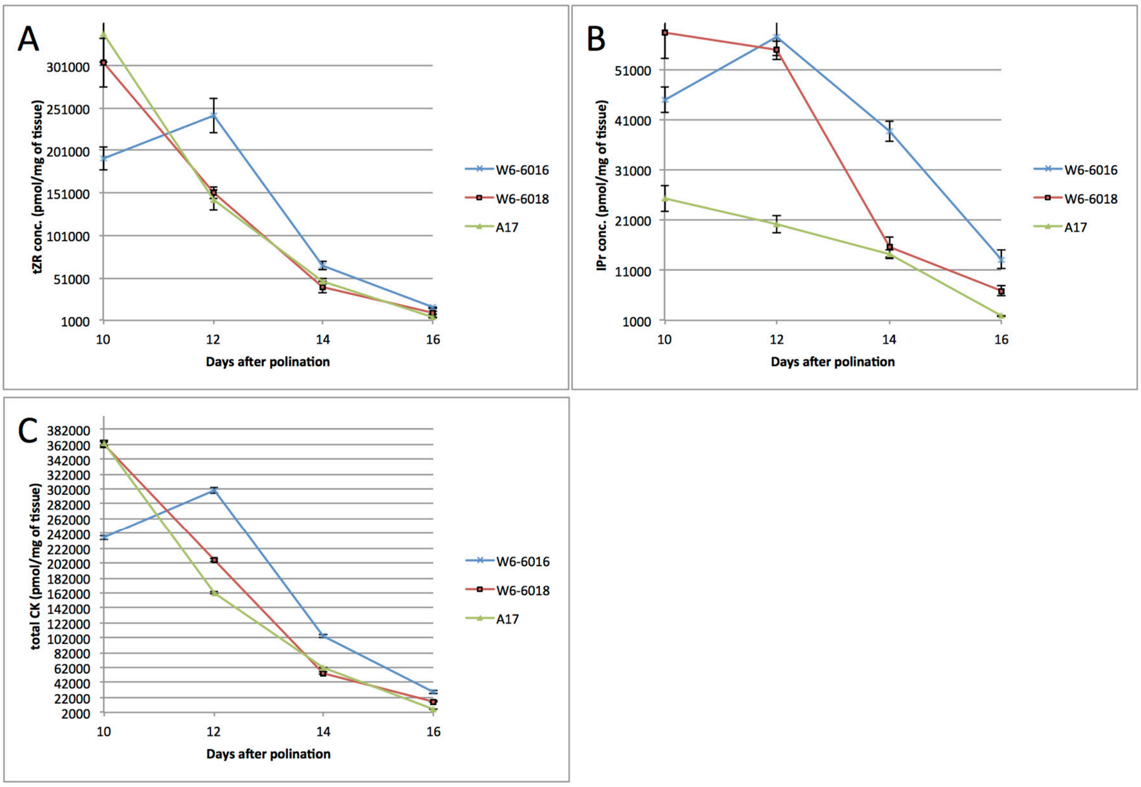

Figure S1. Cytokinines (CK) and IAA-Asp profiles of the three accessions. A-C: different CK concentra2ons in the three accessions (blue lines W6-6016; red lines W6-6018 and green lines creference accession A17). tZR form of cytokinines (**A**); IPr form of cytokinines (**B**); sum of tZR and Ipr forms of cytokinines; (**C**) are plotted against the developmental age (DAP) of the seed.
